# Characterizing individual and methodological risk factors for survey non-completion using machine learning: findings from the U.S. Millennium Cohort Study

**DOI:** 10.1186/s12874-025-02620-3

**Published:** 2025-07-14

**Authors:** Nate C. Carnes, Claire A. Kolaja, Crystal L. Lewis, Sheila F. Castañeda, Rudolph P. Rull, for the Millennium Cohort Study Team, Ania Bukowinski, Felicia Carey, Toni Geronimo-Hara, Clinton Hall, Judy Harbertson, David Ignacio, Isabel G. Jacobson, Cynthia A. LeardMann, Vanessa Perez, Aprilyn Piega, Anna Rivera, Rosa Salvatier, Neika Sharifian, Steven Speigle, Daniel Trone, Javier Villalobos, Katie Zhu

**Affiliations:** 1 Deployment Health Research Department, Naval Health Research Center, San Diego, CA USA; 2 Leidos, Inc, San Diego, CA USA

**Keywords:** Military cohort, Missing data, Survey non-completion, Machine learning

## Abstract

**Background:**

Missing survey data can threaten the validity and generalizability of findings from longitudinal cohort studies. Respondent characteristics and survey attributes may contribute to patterns of survey non-completion, a form of missing data in which respondents begin but do not finish a survey, that can lead to biased conclusions. The objectives of the present research are to demonstrate how machine learning can identify survey non-completion and to characterize individual and methodological factors that are associated with this form of data missingness.

**Methods:**

The present study developed a novel machine learning algorithm to characterize survey non-completion in the Millennium Cohort Study during the 2019–2021 data collection cycle that included a 30- to 45-min paper or web-based follow-up survey for previously enrolled panels (Panels 1–4, *n* = 80,986) and a 30- to 45-min web-based baseline survey for new enrollees (Panel 5, *n* = 58,609). We then examined the effect of individual characteristics and survey attributes on survey non-completion.

**Results:**

This algorithm achieved 99% accuracy and showed that 0.29% of follow-up respondents and 15.43% of new enrollees were survey non-completers. Our findings suggest that certain military and sociodemographic characteristics (e.g., enlisted pay grades) were associated with increased survey non-completion in the 2019–2021 cycle. Survey attributes explained a large proportion of the variability in survey non-completion, with our analyses indicating a higher likelihood of survey non-completion in Sects. (1) located toward the beginning of the survey, (2) with sensitive questions, and (3) with fewer questions.

**Conclusion:**

This research highlights the importance of accounting for potential respondent bias due to survey non-completion and identifies factors associated with this type of missing data.

**Supplementary Information:**

The online version contains supplementary material available at 10.1186/s12874-025-02620-3.

## Background

Longitudinal cohort studies are designed to answer questions regarding prospective relationships between exposures and long-term outcomes using, among other objective and clinical measures, repeated self-report survey data. However, missing data is a common challenge in these studies, with rates of item non-response varying widely depending on the population and study design [1]. Missing survey data may limit the validity of conclusions drawn from such studies, making it more difficult for health cohorts to provide insights about, for example, morbidity or mortality [2]. Thus, it is crucial for researchers to understand the pattern of missing survey data*,*which may occur in three general forms: missing not at random (MNAR), missing at random (MAR), and missing completely at random (MCAR) [2]. MAR occurs when the likelihood of missingness for a given variable is systematically related to other observed characteristics, whereas when data are MNAR, the likelihood of missingness depends directly on the unobserved (i.e., missing) values themselves. MCAR occurs when the probability of missing data is unrelated to both observed and unobserved data (e.g., a participant accidentally skips an item) [2].

Patterns of missing data such as MAR and MNAR can result in biased conclusions because the missing data is systematically related to either observed or unobserved variables, which can distort the true relationships between exposures and outcomes [2, 3]. Missing data in survey research due to survey non-completion may manifest as either MAR or MNAR, each carrying distinct biases and implications for data analysis and interpretation of findings [2]. Although statistical approaches have been developed to mitigate potential biases, the appropriate approach depends on an understanding of missing data patterns and the amount of missing data [4].

Here, “survey non-completion” refers to instances in which enrolled participants initiate but do not complete a baseline and follow-up survey, distinguishing it from general survey nonresponse, missing values, item nonresponse, and other instances of respondent bias.

Factors influencing survey non-completion can fall broadly into two categories: survey attributes (which the study investigators can modify) and participant characteristics (which the study investigators may know). The nature of survey questions, especially when inquiring about sensitive topics (e.g., private behavior, socially undesirable behavior, or stigmatized conditions), has been shown to increase missingness [5]. Historically, questions on employment income were considered sensitive and resulted in higher rates of missingness [6]. Less consistently, the length or complexity of the survey (e.g., survey burden) has been examined as a factor affecting the rate of missing data [4, 7]. Order of survey questions may affect item-level missingness too, such that studies might strategically place lower burden or more salient questions toward the beginning of the survey [8]. Participant characteristics have been less studied and limited to published findings suggesting that those of younger age and lower socioeconomic status may be more likely to have missing data [1, 9, 10]. Survey non-completion resulting from survey attributes versus participants characteristics have different methodological implications for the attenuation of missingness [1]. For example, multiple imputation may be more appropriate when survey non-completion results from survey attributes rather than participant characteristics [2, 11].

The objectives of the present research are to practically demonstrate how to identify survey non-completion and to characterize the risk factors associated with this unique form of data missingness. While research on survey non-completion has been largely conducted in civilian populations, less is known about how survey non-completion manifests in military populations that are generally over-surveyed [12]. Utilizing data from the Millennium Cohort Study (MCS), the largest U.S. prospective military health study whose population has a unique surveying history and sample characteristics, this study developed and validated a machine learning algorithm that differentiates between survey non-completion and other forms of missing data [13]. Leveraging this algorithm, this study then evaluated the effect of individual characteristics (e.g., military and sociodemographic characteristics) and survey attributes (e.g., question sensitivity, item location on the survey, and survey length) on survey non-completion to better understand the reasons why people initiate but do not complete surveys in medical and related fields.

## Methods

### Study population

The present research utilized data from 139,595 participants enrolled in the MCS, the largest and longest running prospective cohort study of current and former U.S. service members, who started (but need not have completed) a survey during the most recent survey cycle (2019–2021). Individuals enrolled into the study were randomly selected from active duty, Reserve, and National Guard Department of Defense administrative rosters to complete a baseline survey in one of five panels (2001–2003, 2004–2006, 2007–2008, 20,011–2013, 2020–2021), with more complex sampling procedures utilized in later panels (e.g., oversampling subgroups of interest). During the most recent data collection period, 80,986 Panel 1–4 participants responded to paper or web-based surveys between August 2019 and August 2021, and 58,609 Panel 5 participants were enrolled and invited to complete the web-based baseline survey between September 2020 and August 2021. Participants were enrolled into Panel 5 if they signed the expanded Health Insurance Portability and Accountability Act (HIPAA) waiver and consent form at the beginning of the survey; the study did not exclude participants on the basis of missing survey data. The study protocol and survey were approved by the Naval Health Research Center Institutional Review Board (protocol NHRC.2000.0007). All participants in this study provided written informed consent at study enrollment.

### Measures

#### Demographic characteristics

Demographic (i.e., sex at birth, age, and race and ethnicity) and military characteristics (i.e., pay grade, service component, service branch, separation from military, deployment history, and length of service) were obtained from the Defense Manpower Data Center (DMDC) as administrative data. Marital status was self-reported on the survey and, if missing, backfilled with DMDC data. All variables were current as of survey completion date for active duty and Reserve/National Guard participants, or last known status for veteran participants.

#### Millennium Cohort Study survey

The survey measures a wide range of variables encompassing sociodemographic factors, military service factors, stressful life events, psychosocial factors, health-related behaviors, physical health, illness and injury, medical conditions, and health care utilization (for a review of survey measures, methodology, and data linkages, see Castañeda et al., 2023). Given that our interest was in missingness, and not the content of these questions per se, all possible survey variables that we would expect every participant to see were included in the analysis, with each participant response converted to a dummy-coded missing value flag. Variables with missingness that would not be diagnostic due to non-exposure in the survey were excluded from the analysis. For new enrollee participants, these excluded variables included skip pattern, study attribute, and follow-up only, resulting in 250 items (see table in Additional file 1). For follow-up participants, these excluded variables included skip pattern, study attribute, new enrollee only, paper or web survey only, and combat exposure variables because many participants were separated from the military; this resulted in 176 items.

#### Survey attributes

Each survey question included in the analysis (for new enrollee participants specifically) was qualitatively coded for sensitivity (or how personal, invasive, threatening, or uneasy the question might be perceived to be by a participant answering the question) independently by two reviewers, with these codes anchored from 0 (low sensitivity) to 2 (high sensitivity). For example, questions on adverse childhood experiences, homelessness, sexual orientation, and life stressors were scored as high sensitivity. These survey questions were then indexed by the order in which they would have appeared to participants (range = [0, 249]) and grouped by survey subsection, indicating the questions that would have appeared together on the survey. This process resulted in 39 survey subsections and permitted the computation of the average question location (*M* = 143.13, *SD* = 80.09), question sensitivity (*M* = 0.44, *SD* = 0.60), and count of questions (*M* = 6.44, *SD* = 5.62) within each section.

### Development of a missingness algorithm

**Fig. 1:**
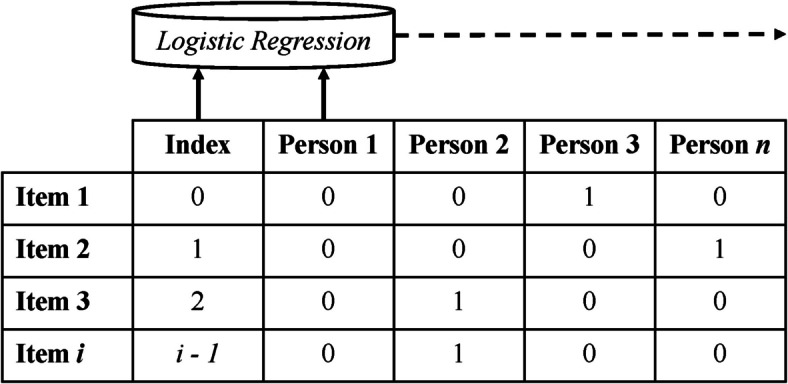
Diagram of algorithm for decomposing MAR and MNAR. Index is item location and person responses are missing value flags (not missing = 0, missing = 1). Logistic regression classifier iterates over each person using index to predict responses. For each person, logistic regression outputs index threshold differentiating missing from not missing. In this example, Person 2 quit the survey after Item 2 (i.e., MNAR) and everyone else completed the survey because they either answered every item or only exhibited MAR item skipping

We developed a machine learning algorithm to decompose MAR and MNAR at an individual level of analysis (see Fig. 1 for a visual diagram of this procedure). We took the ordered data with missing value flags, transposed them as before, and iterated over each person (now represented by a column) to fit a logistic regression classifier. Each of these regressed that person’s missing value flags on the index (or location) of the survey items associated with those missing value flags. This classifier identified the index threshold that differentiates zeroes (not missing) from ones (missing); a threshold at the beginning would indicate a participant who consented and immediately quit thereafter, a threshold at the end would indicate a participant who completed the entire survey, and a threshold in between would indicate a participate who started but quit the survey at some point. We output the classification accuracy from these different classifiers, which revealed that they were, on average, extremely accurate at finding this threshold for both new enrollees (*M* = 0.99, *SD* = 0.019) and follow-ups (*M* = 0.98, *SD* = 0.021). This suggests the machine learning algorithm was effective at identifying and characterizing survey non-completion.

**Fig. 2:**
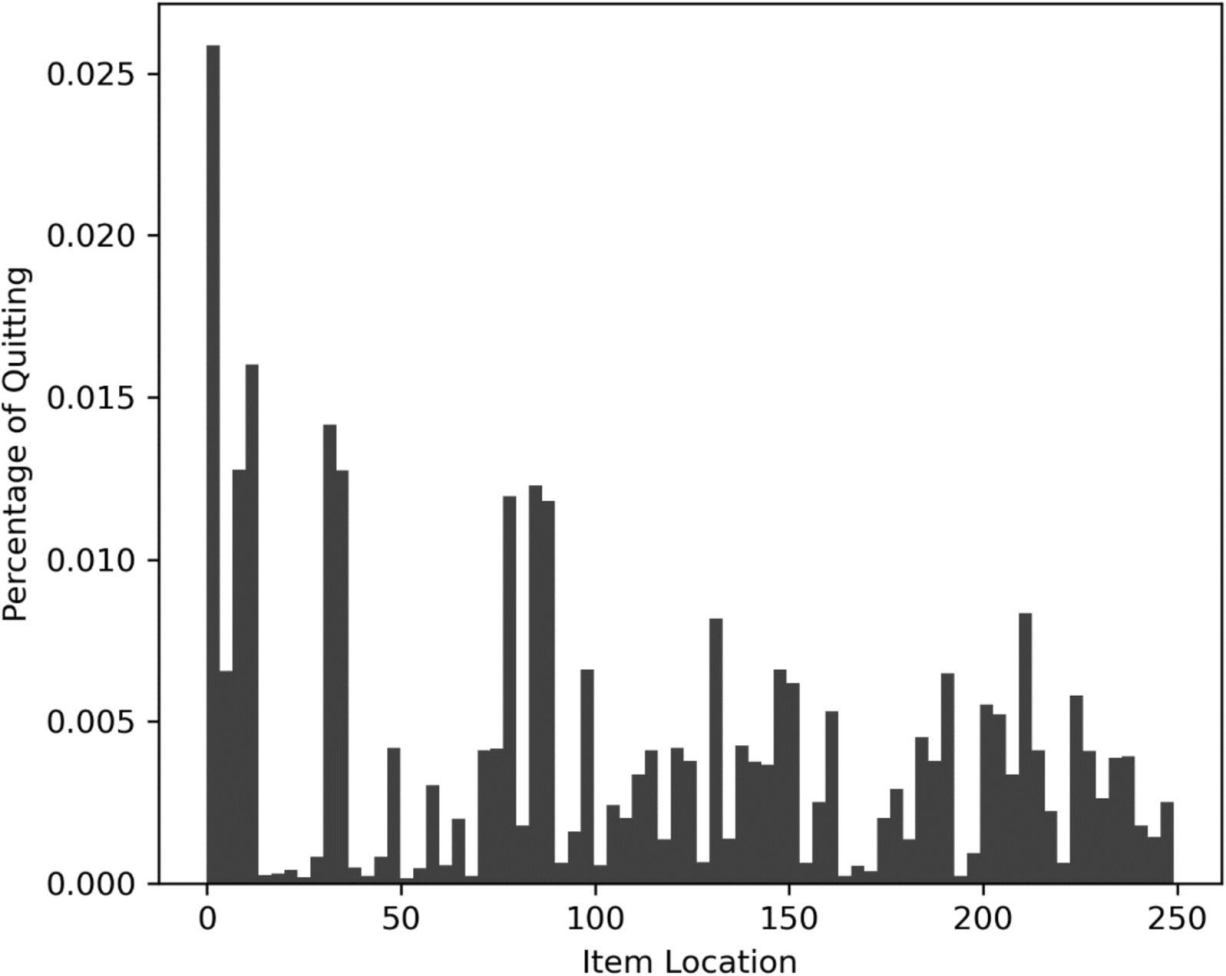
Frequency histogram of quit location among new enrollees. Item location represents the position of an item in the survey, with the first item in the survey having a location of 0 and the last item in the survey having a location of 249

**Fig. 3:**
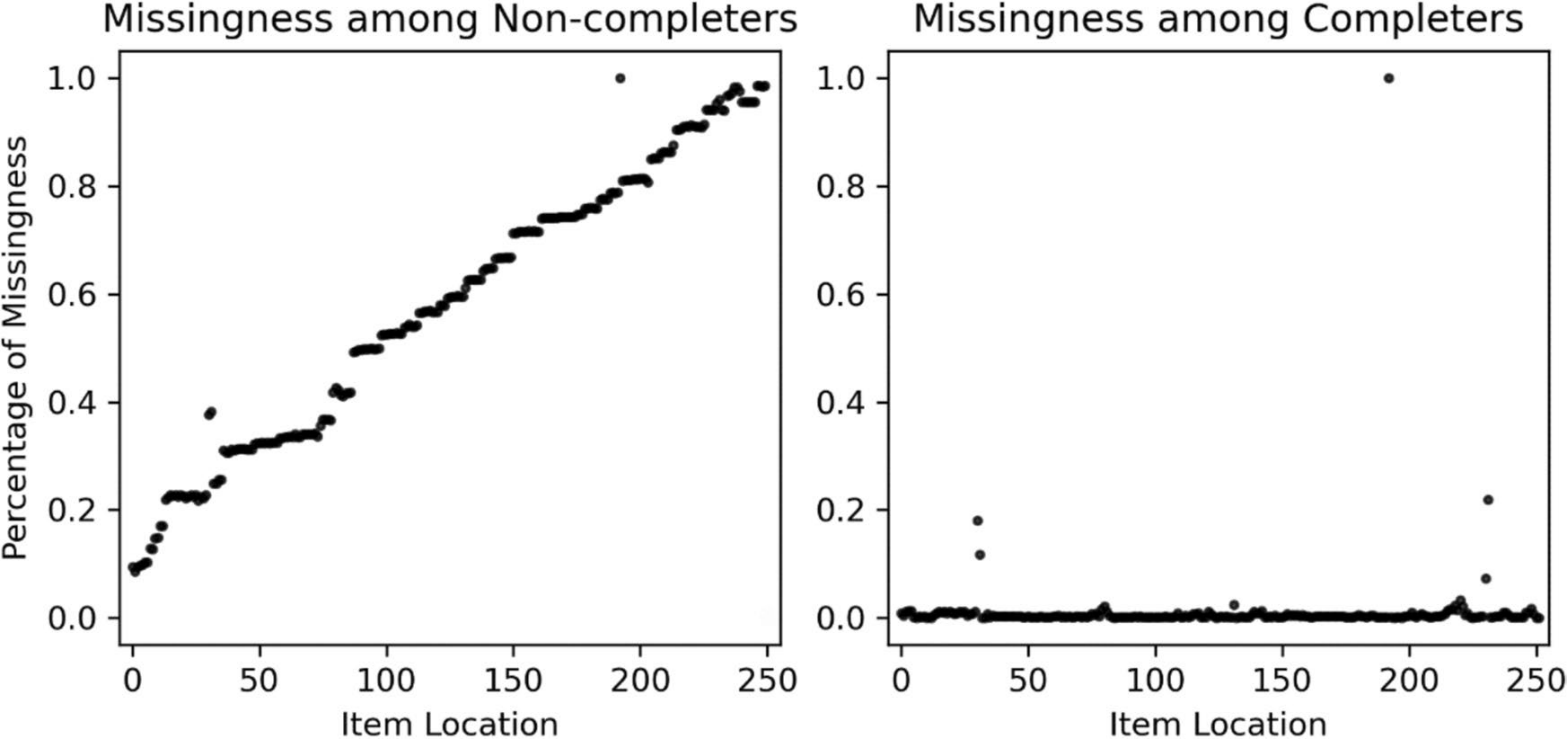
Decomposition of missingness by non-completers versus completers among new enrollees. Item location represents the position of an item in the survey, with the first item in the survey having a location of 0 and the last item in the survey having a location of 249. Non-completers and completers represented those who did or did not quit the survey respectively

This machine learning algorithm was used to identify survey non-completers (vs. completers) and the estimated survey location where they broke off or quit (vs. completed) the survey. We output the predicted values from each of the machine learning classifiers to reconstruct the location where each participant quit (or completed) the survey and identify those who were non-completers (vs. completers). Of the 80,986 follow-ups, only 234 respondents (0.29%) were classified as non-completers. However, of the 58,609 new enrollees, 9,046 (15.43%) were classified as non-completers, which represents a significant difference in non-completion rates between these two groups, *χ*^*2*^(1) = 12,568.17, *p* < 0.001. As seen in Fig. 1, the modal quit location among new enrollees was the beginning of the survey, but there was also significant variability in these quit locations (*M* = 104.14, *SD* = 78.94). As seen in Fig. 2, if we inspect the frequency of missingness by item location among those classified as completers versus non-completers, we can see that these groups successfully decompose missingness into MAR and MNAR, respectively; non-completers show an increasing trend as missingness aggregates from those who have quit the survey, whereas no trend is present among completers who instead exhibit increased missingness on a few well-distributed items (Fig. 3).

### Analytic plan

Descriptive statistics of the demographic and military characteristics among new enrollee and follow-up participants were calculated. Data analysis proceeded in four steps using open-source packages in Python (Python Software Foundation, Beaverton, Oregon, USA). First, the amount and type of missingness in the survey was evaluated, with a specific focus on MAR versus MNAR. Second, the effect of different survey attributes on survey non-completion was tested. Third, the effect of different individual sociodemographic and military characteristics on survey non-completion was tested.

## Results

### Descriptive statistics

Military and sociodemographic characteristics of new enrollee and follow-up survey participants at the time of survey completion for this wave of data collection are shown in Table 1. In both the new enrollee and follow-up samples, a majority of participants were male, White, non-Hispanic, and enlisted pay grade. Compared with follow-up participants, new enrollees were younger (average age of 26 vs. 46 years), more likely to be active duty (85% vs. 47%), and in the Air Force (39% vs. 30%). In addition, new enrollees were less likely to have separated from the military (< 1%) compared with over 29% of follow-ups and less likely to have deployed in support of post-9/11 operations since September 2001 (32% vs. 66%).

**Table 1:** Sociodemographic and military characteristics of new enrollees and follow-ups

	**New enrollee participants** **Panel 5, *n* = 58,609**	**Follow-up participants** **Panels 1–4, *n* = 80,986)**
	***M* (*SD*)**	***M* (*SD*)**
Age, years	26.31 (4.65)	46.30 (11.20)
Length of service, years	3.82 (1.42)	16.57 (6.66)
	%	%
Sex		
Male (vs. female)	69.2	70.3
Marital status		
Single, never married	49.7	9.7
Married	44.4	73.0
Separated	1.9	2.5
Divorced	4.0	13.7
Widowed	0.1	1.1
Race and ethnicity		
American Indian or Alaska Native, non-Hispanic	1.0	1.4
Asian or Pacific Islander, non-Hispanic	9.3	4.1
Black, non-Hispanic	12.8	9.3
Hispanic or Latino	18.9	7.3
Multiethnic or Other, non-Hispanic	2.8	1.0
White, Non-Hispanic	55.2	76.9
Pay grade		
Enlisted (vs. officer)	79.8	68.7
Service component		
Active duty (vs. Reserve/National Guard)	85.0	46.6
Service branch		
Air Force	39.4	29.6
Army	35.3	44.9
Coast Guard	2.9	1.8
Marine Corps	11.0	7.2
Navy	11.4	16.6
Currently separated		
Yes (vs. no)	.7	28.9
Ever deployed		
Yes (vs. no)	31.6	65.8

Age and length of service descriptive statistics represent *M* (*SD*). Currently separated and ever deployed represent participant status as of survey date

#### Tests of missingness

On average, new enrollees exhibited significantly more missingness per person (*M* = 24.67, *SD* = 59.42) than did follow-up responders, *M* = 1.60, *SD* = 4.24, *t*(139,593) = 110.14, *p* < 0.001. For each group, we transposed the ordered data (making items the rows and individuals the columns), summed the number of missing values for each item to construct a time series data structure, and conducted an augmented Dickey-Fuller (ADF) test on this summed missingness indicator to investigate whether it was non-stationary over the course of the survey. Being stationary means that the time series has statistical properties that are constant across each point at which it might be observed. The null hypothesis of this test is that a time series has a unit root and is thus non-stationary, which in this case would indicate that participants’ data are MNAR because they exhibit signals of a trend (i.e., they are time dependent, or in this case, order dependent). For new enrollees, we failed to reject the null hypothesis (ADF = − 2.53, *p* = 0.108) and so their data are MNAR and indicative of a trend. For follow-up respondents, we rejected the null hypothesis (ADF = − 4.16, *p* < 0.001), which indicates their data are MAR and primarily reflect question skipping.

#### Effect of survey attributes on non-completion

We specified a regression model to understand why some new enrollee participants were not completing the survey. This model tests the effect of survey attributes—including question sensitivity, location, and section length—on survey non-completion. The observations in this analysis were the survey subsections themselves, the predictors were survey attributes, and the outcome was the count of those who quit the survey per subsection. We first calculated the average number of missing responses (*M* = 5952.90, *SD* = 2430.70) and number of people quitting the survey (*M* = 231.95, *SD* = 318.72) within each of these subsections to serve as outcome variables. We performed Poisson regression, which utilizes a log link function, because count data generally follow a Poisson distribution. The model regressed the count of non-completions on question sensitivity, location, and count. Importantly, question count was also specified as an exposure variable because subsections with more questions would provide participants with more opportunities to quit, all else being equal.

The Poisson regression model successfully converged and was a good fit to the data (*R*^2^_*pseudo*_ = 0.312). An inspection of the parameter estimates revealed that subsections located earlier in the survey (relative risk [RR] = 0.53, *b* = − 0.64, *z* = − 45.98, *p* < 0.001, 95% CI = [− 0.67, − 0.61]), or composed of more sensitive questions (RR = 1.17, *b* = 0.16, *z* = 11.32, *p* < 0.001, 95% CI = [0.13, 0.18]), or containing fewer questions overall (RR = 0.54, *b* = − 0.61, *z* = − 57.30, *p* < 0.001, 95% CI = [− 0.63, − 0.59]) were associated with a significantly higher relative risk of survey non-completion.

#### Effect of sociodemographic and military characteristics on non-completion

Lastly, we investigated the effect of participant characteristics, including both sociodemographic and military characteristics, on survey non-completion among new enrollees. Unlike the previous analysis, which looked at survey subsections, the observations in this analysis were individual respondents and the outcome was whether or not each individual was a non-completer. We performed logistic regression, which utilizes a logit link function, because the outcome was dichotomous. The model regressed non-completion on sex at birth, age, marital status, race and ethnicity, pay grade, component, service branch, separation from the military, deployment history, and length of service; categorical variables were dummy coded and continuous variables were min–max normalized. The data were randomly split into a training set (75%) and a test set (25%), and the model was fit to the training data using L1 regularization (*α* = 10). The model successfully converged and yielded modest but acceptable fit to the data (*R*^2^_*pseudo*_ = 0.02). The training set (*A*_*B*_ = 0.58) and test set (*A*_*B*_ = 0.58) achieved similar, balanced accuracy scores using the same fitted model, suggesting we did not overfit to the data. The parameter estimates from the training set are shown in Table 2.

**Table 2:** Estimated effects of sociodemographic and military characteristics on survey non-completion

	***OR* (95% CI)**	***p***
Intercept	.22 (.29,.24)	<.001
Age, years	1.00 (.71, 1.40)	1.000
Length of service	1.00 (.73, 1.37)	1.000
Female sex (ref: male)	1.09 (1.03, 1.16)	.002
Marital status (ref: single, never married)		
Divorced	.62 (.53,.72)	<.001
Married	.77 (.73,.82)	<.001
Separated	.15 (.10,.21)	<.001
Widowed	1.00 (.32, 3.08)	1.000
Race and ethnicity (ref: White, non-Hispanic)		
American Indian or Alaska Native, non-Hispanic	1.00 (.76, 1.31)	1.00
Asian or Pacific Islander, non-Hispanic	1.09 (.99, 1.20)	.068
Black, non-Hispanic	1.73 (1.60, 1.86)	<.001
Hispanic or Latino	1.25 (1.17, 1.34)	<.001
Multiethnic or Other, non-Hispanic	1.28 (1.09, 1.50)	.002
Officer pay grade (ref: enlisted)	.66 (.61,.72)	<.001
Reserve/National Guard (ref: active duty)	.74 (.68,.80)	<.001
Service branch (ref: Army)		
Air Force	.78 (.74,.83)	<.001
Coast Guard	.76 (.64,.91)	.002
Marine Corps	1.01 (.92, 1.10)	.913
Navy	.93 (.85, 1.02)	.129
Currently separated (ref: no)	.68 (.47,.98)	.037
Ever deployed (ref: no)	1.09 (1.03, 1.16)	.004

Age and length of service were centered at the minimum. The outcome variable was survey non-completion (i.e., whether or not each respondent failed to complete the survey)

CI, confidence interval; OR, odds ratio

Among sociodemographic characteristics, adjusted analyses revealed significant associations of an at least minimally practical magnitude (*OR* ≥ 1.49 or ≤ 0.67) [14] between both marital status and race and ethnicity with the likelihood of survey non-completion. Compared with single participants, those who were married, divorced, or separated were more likely to complete the survey. Compared with White non-Hispanic service members, Black non-Hispanic service members were more likely to be survey non-completers.

Among military characteristics, we found significant associations of an at least minimally practical magnitude between separation status, pay grade, and, to a lesser extent, both service component and service branch. Participants who had separated from the military, were officers, were Reservists or National Guardsmen, or were members of either the Air Force or Coast Guard were more likely to complete the survey (and relatively less likely to be non-completers than their relevant reference groups). More generally, however, most of the effects of sociodemographic or military characteristics on survey non-completion were small in magnitude and exhibited substantial individual variability.

## Discussion

The present research investigated the incidence and correlates of survey non-completion among current and former U.S. service members enrolled in the MCS. Analyses revealed minimal missingness and little evidence of survey non-completion among follow-up survey respondents, likely reflecting some degree of selection for those who completed one or more earlier surveys. In contrast, new enrollees exhibited higher levels of missingness and robust evidence of survey non-completion, suggesting a unique response pattern among some participants during the initial data collection process. The 15% non-completion rate and pattern of non-completion reported in this study aligns with multiple studies, including two large reviews of 185 opt-in web surveys and 250,000 non-probability surveys conducted through SurveyMonkey, that reported breakoff rates between 12 and 17% and several that found participants break off at the beginning of a survey [15–19]. It is important to understand the characteristics of these non-completing individuals so that selection in the study sample can be evaluated and potentially addressed in future research via appropriate sample weighting. It is also important to understand the potential risk factors for survey non-completion so that the rate of survey non-completion can be attenuated in future data collection procedures through the implementation of methodological changes.

Toward these ends, the present research first developed a novel algorithm for identifying individuals who were survey non-completers and the location at which they quit the survey; this algorithm was approximately 99% accurate. Data contain both responses MAR and MNAR, which means that an arbitrary cutoff based on the proportion of missingness may misspecify the true rate of non-completion. Indeed, this algorithm estimated that the rate of non-completion was 15.43% among new enrollees, whereas a requirement of less than 15% missingness yielded an estimated non-completion rate of 13.95%, suggesting that this simple cutoff underestimated the true non-completion rate by approximately 10%. We then leveraged the non-completion indicators derived from this algorithm, including who and where in the survey enrolled participants quit, to investigate the individual characteristics and survey attributes associated with survey non-completion.

It is somewhat remarkable that the model examining the effect of survey attributes explained greater than 31% of the variability in survey non-completion, as this suggests that survey attributes alone—a factor the researcher controls—represent an important influence over whether a participant does or does not answer the question at hand; this provides further evidence that the question shapes the answer, even if that answer is simply a blank response [20]. An inspection of model coefficients revealed that early survey sections and sensitive questions are critical points where participants are more likely to quit. This suggests that how a survey makes a participant feel upon their initial encounter with it is particularly important, and that perhaps participants may not quit if they can just be nudged in little ways to progress further. Unsurprisingly, people are likely to quit when answering sensitive questions, reinforcing the importance of asking such questions thoughtfully and later in the survey. Lastly, and somewhat surprisingly, participants are more likely to quit in shorter survey sections once one accounts for the confound of exposure, suggesting that including many short sections may be perceived as more onerous than fewer long sections. While these findings provide new insights, they are drawn from correlational data, necessitating caution in making causal inferences or generalizing beyond the sampled population.

The model representing the effect of individual characteristics only explained greater than 2% of the variability in survey non-completion, connoting that sociodemographic and military characteristics are not particularly strong factors for survey non-completion. That said, certain groups, including female, Black non-Hispanic, and Hispanic individuals, were more likely to be survey non-completers, echoing findings in other work that indicate variation in missing data between different groups [21]. This indicates that the risk of selection bias needs to be taken seriously by study planners to ensure the study population is representative. Analyses also indicated that members of the Army or Marine Corps, active component service members, enlisted service members, and those not separated from the military were the most likely to be survey non-completers. This too may represent selection bias that ought to be addressed if future research is to be representative of service members who are part of one or more of these groups.

Together, our findings suggest that survey attributes may be a stronger selection force in shaping non-completion rates than individual characteristics in the MCS and potentially other cohort studies; while not necessarily comparable given the different types of data used, survey attributes explained 13 times more variance than participant characteristics in our analyses. The fact that there was weak selection for non-completion on the basis of individual characteristics supports the generalizability of the MCS and indicates that attenuating non-completion in future work primarily comprises an endeavor of survey design. It is important to note that the survey non-completion observed among Panel 5 MCS participants may be partially an artifact of the survey methods utilized at enrollment. This was the first panel for which the study solely used web-based surveys as the study transitions to predominately web-based survey collection. One possibility is that an increased rate of smartphone use to complete the survey among this younger cohort may have simultaneously made the study more accessible and impacted the completeness of data. Although not examined for this project, prior work suggests that the use of smartphones to complete web surveys may result in increased missingness [22–24]. The use of a novel machine learning approach has allowed for a more nuanced understanding of survey non-completion. Given the distinctive characteristics of military populations, this study underscores the need for further exploration into methodological and individual differences that influence survey non-completion, particularly among oversampled study populations. This may create opportunities for future research in targeted methodological enhancements, design of future surveys, and analyses examining missingness in association to a range of outcomes. For example, it is plausible that the questions respondents choose not to answer, that is missingness itself, can yield meaningful insights about the characteristics of those respondents. Future research could explore whether specific, latent patterns of non-response and non-completion—characterized using algorithms—can predict methodological (e.g., socially desirable responding) or outcome (e.g., psychological health) constructs of interest.

An additional survey design element that may have contributed to the survey non-completion observed among Panel 5 MCS participants was the decision to enroll all Panel 5 participants who signed the consent form and HIPAA waiver. Although this may not align with other national cohort studies that require complete survey data (with varying definitions), it is advantageous for several reasons. External administrative data sources, including deployment, medical, mortality, and suicide records, can be examined for all participants, regardless of completeness of survey data, allowing for inclusion in many studies examining rare or late-onset outcomes. In addition, because the study has access to administrative military records, demographic and military factors are captured regardless of survey responses and this information can be used to weight respondent self-report to the entire enrolled panel or for use in adjusted models. Lastly, all Panel 5 participants were invited to complete the first follow-up survey (which launched in 2024), so self-reported data may be provided later.

Additional limitations of the present research should be considered. The machine learning algorithm utilized depends on a common set of items administered in a common presentation order. This algorithm cannot, at present, account for complex skip logic or randomization within or between survey sections, which may limit its feasibility or accuracy in other contexts. Similarly, the sample of new enrollees from which inferences were drawn may not generalize to other research populations of interest. It is possible that different individual or methodological risk factors may be of greater or lesser importance in other survey populations.

The present research sheds light on the individual characteristics and survey attributes associated with survey non-completion, and it developed an algorithm that can be readily implemented in related research efforts to characterize survey non-completion. These findings can be used to inform future research and guide methodological design of survey operations that could incorporate strategies to reduce drop off during the new enrollee survey. For example, future surveys of new enrollees could include more salient or engaging questions for this population, such as those concerning military experiences, at the beginning of the survey to motivate the substantive number of participants breaking off at the start of the survey. Alternatively, future surveys could oversample sociodemographic groups inversely proportional to their tendency to be survey non-completers to attenuate the effects of selection bias. In addition, future research should investigate other individual characteristics and their association with survey non-completion, such as care observed in medical record data, or deployment experiences. These advancements are vital for enhancing the representativeness and reliability of research findings, ultimately informing better policy and practice in medical applications.

## Supplementary Information

Supplementary Material 1. 2020–2021 new enrollee survey constructs.

## Data Availability

The data that support the findings of this study are not currently publicly available because of institutional regulations protecting service member survey responses, but they are available on reasonable request and require a data use request. The data use request would need to be approved by the NHRC HIPAA Privacy Officer and ensure that the dataset is truly de-identified based on Safe Harbor and expert determinations. Requests for data access may be sent to usn.point-loma.navhlthrschcensan.mbx.nhrc-millennium-cohort-pi@health.mil.

## Abbreviations

HIPAA: Health Insurance Portability and Accountability Act
MAR: Missing at random
MCAR: Missing completely at random
MCS: Millennium Cohort Study
MNAR: Missing not at random
RR: Relative risk
