# Supplementary material for: Characterizing individual and methodological risk factors for survey non-completion using machine learning: findings from the U.S. Millennium Cohort Study
Source: BMC Med Res Methodol. 2025 Jul 14;25:174. doi: 10.1186/s12874-025-02620-3 (PMC12261820; doi:10.1186/s12874-025-02620-3)
Supplement: Supplementary file 1 — Supplementary Material 1. 2020–2021 new enrollee survey constructs. [file 12874_2025_2620_MOESM1_ESM.docx]

**Supplemental Table.** 2020–2021 new enrollee survey constructs

|  | **Number of items** |  |
| --- | --- | --- |
| **Sleep quality** (9 items) | | |
| Duration and medications | 2 |  |
| Insomnia symptoms^a^ | 7 |  |
| **Physical health** (65 items) | | |
| Days missed work, hospitalized | 2 |  |
| Joint pain (i.e., knee, back) | 2 |  |
| General symptoms | 18 |  |
| Sedentary behavior | 1 |  |
| Height and weight | 3 |  |
| Medication (i.e., pain) | 1 |  |
| Somatic symptoms^b^ | 12 |  |
| Medical diagnoses | 26 |  |
| **Well-being** (58 items) | | |
| Quality of life^c^ | 13 |  |
| Posttraumatic stress disorder^d^ | 20 |  |
| Anger^e^ and frequency | 6 |  |
| Depression^b^ | 8 |  |
| Disordered eating^b^ | 2 |  |
| Fast food consumption | 1 |  |
| Generalized anxiety^b^ | 7 |  |
| Medication use^b^ | 1 |  |
| **Support and coping** (29 items) | | |
| Perceived social support^f^ | 6 |  |
| Mental health care utilization | 5 |  |
| Self-mastery^g^ | 7 |  |
| Posttraumatic growth | 11 |  |
| **Military service** (23 items) | | |
| Combat experiences | 14 |  |
| Morale and unit cohesion | 3 |  |
| Military satisfaction | 6 |  |
| **Life experiences** (33 items) | | |
| Discrimination, bullying, harassment, and hazing | 4 |  |
| Risk items^b^ | 4 |  |
| Life stressors | 12 |  |
| Adverse childhood experiences | 4 |  |
| Head injury | 5 |  |
| Motor vehicles | 1 |  |
| Time away from home | 1 |  |
| Homelessness | 2 |  |
| **Alcohol and tobacco use** (13 items) | | |
| Tobacco and vaping use | 7 |  |
| Alcohol use | 6 |  |
| **Family and relationships** (10 items) | | |
| Household composition | 2 |  |
| Marital status | 1 |  |
| Number of children | 1 |  |
| Sexual orientation, attraction, partners | 3 |  |
| Gender identity | 2 |  |
| Attempted pregnancy | 1 |  |
| **Demographics** (10 items) | | |
| Access (e.g., health insurance) | 6 |  |
| Financial status (i.e., income, financial situation) | 2 |  |
| Twin | 1 |  |
| Employment | 1 |  |

Superscript letters indicate standardized measure used for associated items. Number of items in each section provided in parentheses.

^a^ Insomnia Severity Index.

^b^ Patient Health Questionnaire.

^c^ Short Form Health Survey.

^d^ PTSD Checklist for DSM-5.

^e^ Dimensions of Anger Reactions.

^f^ Multidimensional Scale of Perceived Social Support.

^g^ Pearlin Mastery Scale.
